# The Cell Tropism of Porcine Respiratory Coronavirus for Airway Epithelial Cells Is Determined by the Expression of Porcine Aminopeptidase N

**DOI:** 10.3390/v12111211

**Published:** 2020-10-23

**Authors:** Ju-Yi Peng, Darsaniya Punyadarsaniya, Dai-Lun Shin, Suvarin Pavasutthipaisit, Andreas Beineke, Guangxing Li, Nai-Huei Wu, Georg Herrler

**Affiliations:** 1 Institute of Virology, University of Veterinary Medicine Hannover, 30559 Hannover, Germany; Ju-Yi.Peng@tiho-hannover.de (J.-Y.P.); dai-lun.shin@tiho-hannover.de (D.-L.S.); 2 Virology and Immunology Department, Faculty of Veterinary Medicine, Mahanakorn University of Technology, Bangkok 10100, Thailand; darsaniya_p@yahoo.de; 3 Department of Pathology, University of Veterinary Medicine Hannover, 30559 Hannover, Germany; Suvarin.Pavasutthipaisit@tiho-hannover.de (S.P.); andreas.beineke@tiho-hannover.de (A.B.); 4 Department of Pathology, Faculty of Veterinary Medicine, Mahanakorn University of Technology, Bangkok 10100, Thailand; 5 College of Veterinary Medicine, Northeast Agricultural University, 59 Mucai Street, Xiangfang District, Harbin 150000, China; ligx@neau.edu.cn; 6 Department of Veterinary Medicine, National Taiwan University, Taipei 106, Taiwan

**Keywords:** porcine respiratory coronavirus, porcine aminopeptidase N, air–liquid interface culture, tropism

## Abstract

Porcine respiratory coronavirus (PRCoV) infects the epithelial cells in the respiratory tract of pigs, causing a mild respiratory disease. We applied air–liquid interface (ALI) cultures of well-differentiated porcine airway cells to mimic the respiratory tract epithelium in vitro and use it for analyzing the infection by PRCoV. As reported for most coronaviruses, virus entry and virus release occurred mainly via the apical membrane domain. A novel finding was that PRCoV preferentially targets non-ciliated and among them the non-mucus-producing cells. Aminopeptidase N (APN), the cellular receptor for PRCoV was also more abundantly expressed on this type of cell suggesting that APN is a determinant of the cell tropism. Interestingly, differentiation-dependent differences were found both in the expression of pAPN and the susceptibility to PRCoV infection. Cells in an early differentiation stage express higher levels of pAPN and are more susceptible to infection by PRCoV than are well-differentiated cells. A difference in the susceptibility to infection was also detected when tracheal and bronchial cells were compared. The increased susceptibility to infection of bronchial epithelial cells was, however, not due to an increased abundance of APN on the cell surface. Our data reveal a complex pattern of infection in porcine differentiated airway epithelial cells that could not be elucidated with immortalized cell lines. The results are expected to have relevance also for the analysis of other respiratory viruses.

## 1. Introduction

Coronaviruses are responsible for a large number of respiratory tract infections in humans and animals [1]. The porcine respiratory coronavirus (PRCoV) is highly prevalent in swine farms in many countries [2,3,4]. PRCoV is an enveloped, positive-stranded RNA virus belonging to the Alphacoronavirus 1 species within the genus *Alphacoronavirus* in the family *Coronaviridae* [5]. The pulmonary pathogenesis of PRCoV in pigs resembles that of severe acute respiratory syndrome coronavirus (SARS-CoV) in humans in many aspects [6,7]. Both viruses have the same tropism in the respiratory tract, cause bronchointerstitial pneumonia, and replicate for long periods in the lungs [7]. Although most PRCoV infections are mild or subclinical in pigs, it is wildly accepted that PRCoV is an important pathogen contributing to the porcine respiratory disease complex [8]. Therefore, it is imperative to understand the interaction between PRCoV and the respiratory tract.

To elucidate the host–pathogen interactions, we cultured the porcine airway epithelial cells under air–liquid interface (ALI) conditions. These ALI cultures of well-differentiated respiratory epithelial cells are the appropriate model to study the viral infection under conditions that are close to the situation in nature [9]. The ALI cultures contain ciliated cells, mucus-producing cells, secretory cells (club cells), and basal cells [10]. ALI cultures have previously been shown to be superior to the standard cell lines in the analysis of different coronaviruses: HCoV-HKU1, HCoV-229E, and SARS-CoV-2 [11,12]. Furthermore, the porcine ALI cultures have been used to investigate other swine respiratory pathogens [13]. In general, this in vitro model resembles the in vivo situation of the porcine airway epithelium both morphologically and functionally [11,13].

PRCoV uses APN as a receptor to attach to target cells and initiate infection [14,15,16]. APN is a 150 kDa type II transmembrane glycoprotein. APN is expressed in a variety of tissues, including cells of the granulocyte and monocyte lineage, epithelial cells from the intestinal brush border and the respiratory tract [17,18]. Previously, human aminopeptidase N (hAPN) has been reported predominantly expressed on non-ciliated cells in the human bronchial epithelial cells; infection by and replication of human coronavirus 229E (HCoV-229E) has also been shown to occur in non-ciliated cells [14,17]. Such information is not available for PRCoV and porcine aminopeptidase N (pAPN).

Here, we aimed (i) to characterize the infection of differentiated airway epithelial cells by PRCoV, (ii) to identify the cell type susceptible to infection, and (iii) to elucidate whether the distribution of virus receptors determines the cell tropism of the virus. We found that PRCoV infects a subpopulation of the epithelial cells that are not ciliated and do not produce mucus. The cellular receptor for PRCoV, pAPN, is most abundantly expressed on the surface of these non-ciliated cells. This finding is consistent with the notion that pAPN is a major determinant of the cell tropism of this virus. We also report the novel observation that PRCoV infection of porcine airway epithelial cells is dependent on the state of differentiation. Our findings provide new insights into the host-virus interactions of PRCoV that are expected to have relevance also for other coronaviruses.

## 2. Materials and Methods

### 2.1. Porcine Airway Epithelial Cell Cultures

Primary porcine tracheal epithelial cells (PTECs) and primary porcine bronchial epithelial cells (PBECs) were harvested from the 5-month-old pigs’ trachea and bronchial, respectively, as previously described [19,20]. Briefly, PTECs and PBECs firstly maintained in bronchial epithelial cell growth medium (Lonza, Basel, Switzerland). When cell monolayers had reached a confluence of about 80%, cells were transferred to Transwell^®^ (Corning, New York, NY, USA) at a density of 4 × 10^5^ cells per filter and maintained with ALI medium. After the cells reached confluence, the cells were maintained under air–liquid interface conditions for at least 3 weeks at 37 °C in a humidified 5% CO_2_ atmosphere. The cells were tested negative for porcine-specific respiratory tract pathogens.

### 2.2. Cell and Virus

Swine testicular (ST) cells were maintained in Eagle’s minimal essential medium (EMEM; PAN, Bavaria, Germany) supplemented with 10% fetal calf serum. The cells were incubated in a humidified atmosphere containing 5% CO_2_ at 37 °C and passaged every 2 to 3 days. PRCoV Bel85 was obtained from Luis Enjuanes [20]. The virus stock was propagated on ST cells in EMEM. After incubation for 36 to 48 h at 37 °C, the supernatants were harvested, centrifuged, and stored at −80 °C.

### 2.3. Virus Infection of Differentiated Epithelial Cells

Virus infection of differentiated epithelial cells is performed as previous described [13]. Briefly, the total amounts of cells in each well is approximately 5 × 10^5^. The cells were inoculated by PRCoV from the apical side at 1 × 10^3^ focus forming units (FFU). After 2 h of incubation at 37 °C, the cells were rinsed with PBS twice to remove unbound viral particles and fresh ALI medium was added. At different time points, 100 μL of EMEM was added to the apical surface and the cultures were incubated for 30 min at 37 °C. The harvests were collected at different times post-virus-infection and the viruses were titrated by focus forming assay on ST cells. For immunofluorescence analysis, infected PTECs and PBECs were fixed with 3% paraformaldehyde (PFA).

### 2.4. Immunohistochemistry Staining (IHC) and Immunofluorescence Analysis (IFA)

To localize the expression of pAPN, IHC staining of porcine tissue and PTECs was applied [21]. Briefly, the samples were fixed with 3% paraformaldehyde (PFA) in PBS for 1 h. Samples were washed with phosphate-buffered saline containing 5% skim milk and embedded in paraffin blocks for sectioning. Thin paraffin serial sections of 3–4 μm were made using a rotary microtome. For staining purposes, samples were deparaffinized, rehydrated, and followed by blocking endogenous peroxidase activity with 0.5% H_2_O_2_ diluted in ethanol. Nonspecific binding sites were blocked with normal goat serum. Subsequently, the sample was incubated with a rabbit polyclonal anti-pAPN antibody (1:800) at 4 °C overnight as previously report [22]. Then, samples were incubated with biotinylated goat-anti-rabbit IgG diluted 1:200 (BA-1000, Vector Laboratories, Burlingame, CA, USA) was used as secondary antibody for 45 min at room temperature. Subsequently, the slides were incubated with the peroxidase-conjugated avidin-biotin-complex (ABC method, PK-6100, VECTASTAIN Elite ABC Kit; Vector Laboratories) for 30 min at room temperature. The positive antigen–antibody reactions were visualized by incubation with 3,3′-diaminobenzidine tetrahydrochloride (DAB, Sigma-Aldrich, Missouri, USA) and H_2_O_2_ followed by counterstaining with Mayer’s hematoxylin (Merck).

To analyze the cell tropism of PRCoV, IFA was performed as previously described [13]. Briefly, the samples were fixed with 3% PFA for 1 h and permeabilized with 0.5% Triton X-100 for 20 min at room temperature. Samples were further blocked with 5% goat serum and 1% bovine serum albumin. The coronavirus nucleocapsid protein was stained by a monoclonal mouse anti-coronavirus-antibody (FIPV3-70; 1:1000, Invitrogen, Carlsbad, CA, USA), and followed by incubation with fluorescent secondary antibody (1: 1,000; Alexa Fluor 488 anti-mouse IgG [H + L] antibody, Thermo Fisher, Massachusetts, USA). Ciliated cells were visualized using a Cy3-labeled monoclonal antibody against β-tubulin (1:300; Sigma-Aldrich, St. Louis, MO, USA). Mucus-producing-cells were stained by biotinylated anti-mucin-5AC antibody (1:250; Abcam, Cambridge, England), and followed by incubation with Streptavidin−Cy3™ from *Streptomyces avidinii* (1:1000, Merck, Darmstadt, Germany). For detection of pAPN, the samples were stained without the permeabilization. The expression of pAPN was stained by rabbit anti-pAPN antibody, following by Alexa Fluor 488 anti-rabbit IgG [H + L] antibody (Life Technologies, Carlsbad, CA, USA). The nuclei were stained by 1 μg/ml DAPI (4′,6-diamidino-2-phenylindole), and embedded with ProLong^®^ Gold Mountant (Life Technologies, Carlsbad, CA, USA). Immunofluorescence analysis was performed using a Nikon Eclipse Ti microscope and NIS Elements AR software (Nikon, 4.11,.0, Tokyo, Japan), or using a TCS SP5 confocal laser scanning microscope equipped with a 63 × (NA, 1.40) oil HCX PL Apo objective (Leica, Mannheim, Germany). ImageJ/Fuji software calculated the expression amount of pAPN.

### 2.5. Virus Titration

To determine the infectivity of the harvested supernatants, a focus-forming assay was performed as described previously with some modifications [19]. Briefly, ST cells were seeded in 96-well plates one day before the experiment. Serial 10-fold dilutions of harvests from ALI cultures were performed and then inoculated on ST cells for 1 h at 37 °C. Cells were overlaid with Avicel. After incubation for 24 h at 37 °C, cells were fixed with 3.7% formalin, and permeabilized with quencher buffer (0.5% Triton X-100 with 20 mM glycine in PBS). A primary anti-coronavirus-antibody (FIPV3-70; 1:1000, Invitrogen, Carlsbad, CA, USA) was added for 1 h at room temperatures, followed by a secondary horseradish peroxidase (HRP) antibody (KPL) for 1 h at room temperature. Subsequently, a substrate (True Blue; KPL, Kingston, MA, USA) was used for immunological staining. The calculated virus titer is indicated in FFU/ml.

### 2.6. Statistical Analyses

If not stated otherwise, experiments were performed at least six times with samples derived from three donors. Results are expressed as the means with standard error of the mean (SEM). Data were analyzed by one-way ANOVA with Tukey’s post-hoc test, using GraphPad Prism (GraphPad Software version 5, San Diego, CA, USA) software.

## 3. Results

### 3.1. Replication of PRCoV on wdPTECs

**Figure 1:**
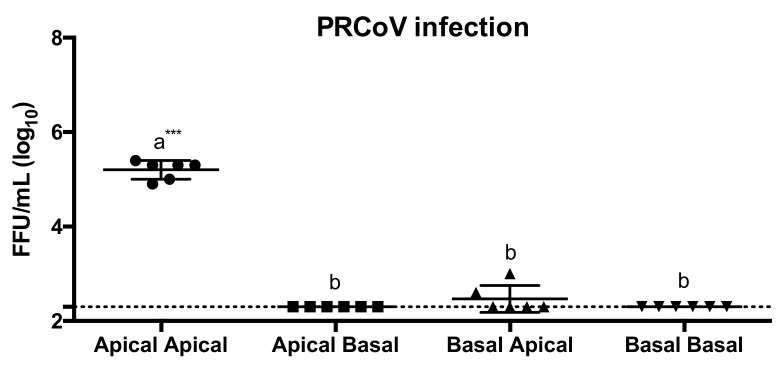
Entry and release of PRCoV Bel85 on well-differentiated porcine tracheal epithelial cell (wdPTECs). PRCoV Bel85 was used to infect wdPTECs an 1 × 10^3^ FFU, and 96 h later, the apical and the basolateral medium were collected for focus-forming assay in ST cells. The results were shown as means ± SEM of six wdPTECs from three independent donors. The dashed line was the detection limit for the foci-forming assay. Statistical analysis was performed by one-way ANOVA and followed by Tukey’s multiple comparisons test. a, b significant differences between groups are indicated with different letters (*** *p* < 0.001).

To characterize the infection of porcine airway epithelial cells by PRCoV, we determined the site of virus entry and virus release in well-differentiated porcine tracheal epithelial cells (wdPTECs). Cells that had been differentiated on filters were infected from either the apical or basolateral side. At daily intervals post-infection, the media from the apical and basolateral compartments were analyzed for the presence of infectious PRCoV. As shown in Figure 1, both virus entry and virus release occurred predominantly via the apical plasma membrane domain.

### 3.2. Cell Tropism of PRCoV

**Figure 2:**
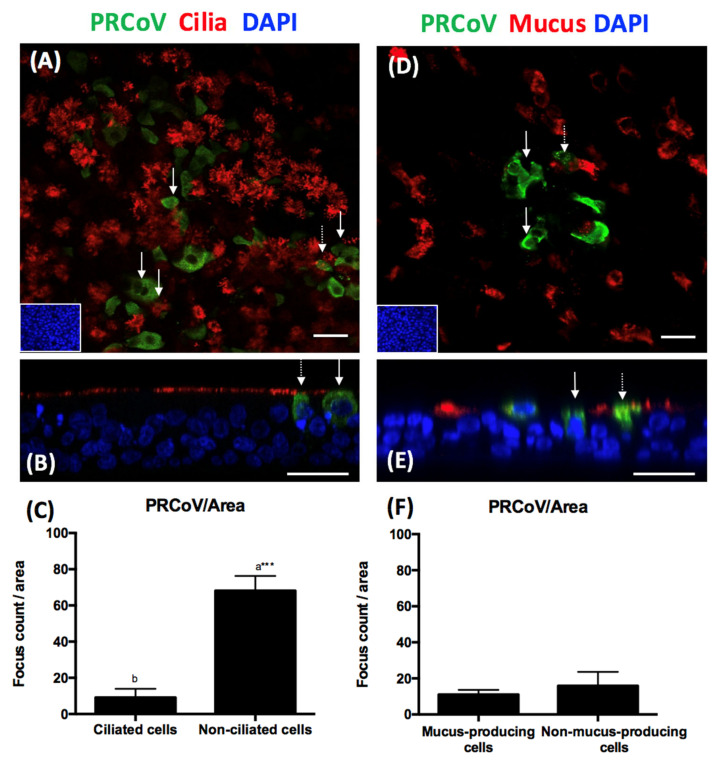
Tropism of PRCoV Bel85 to distinct types on well-differentiated porcine tracheal epithelial cells (wdPTECs). WdPTECs were infected with PRCoV from the apical side an 6 × 10^4^ FFU/ml and fixed at 1 dpi. (**A**,**B**) presence of PRCoV nucleocapsid (green) mainly in non-ciliated cells (solid arrow) and rarely in ciliated cells (red, dash arrow). (**C**) quantification of PRCoV antigen on the surface of ciliated cells and non-ciliated cells was calculated by ImageJ. (**D**,**E**) presence of PRCoV nucleocapsid (green) mainly in non-mucus-producing cells (solid arrow) and rarely in mucus-producing cells (red, dash arrow). (**F**) Quantification of PRCoV antigen on the surface of mucus-producing cells and non-mucus-producing cells was calculated by ImageJ. The results were shown as means ± SEM of six wdPTECs from three independent donors. Statistical analysis was performed by a Student’s *t*-test. a, b significant differences between groups are indicated with different letters (*** *p* < 0.001). Scale bars, 25 μm (**A**,**D**). Scale bars, 50 μm (**B**,**E**).

To elucidate the cell tropism of PRCoV, we infected wdPTECs with PRCoV. At one-day post-infection, the cells were fixed with 3% PFA and stained for the presence of virus antigen, cilia (β-tubulin) and/or mucins (mucin-5AC). The result of the co-localization anaylsis by confocal immunofluorescence microscopy is shown in Figure 2. Among wdPTECs, virus antigen was predominantly detected in non-ciliated cells; only a few ciliated cells were infected by PRCoV. Among the non-ciliated cells, virus antigen was mainly detected on non-mucus-producing cells; only a few of the mucus-producing cells were infected by PRCoV. Infection of wdPTECs did not have any detrimental effect on the cell integrity. Both the morphology and the ciliary activity were unaffected when compared to control cultures.

### 3.3. Expression of pAPN on the Porcine Trachea and Well-Differentiated Porcine Tracheal Epithelial Cells

**Figure 3:**
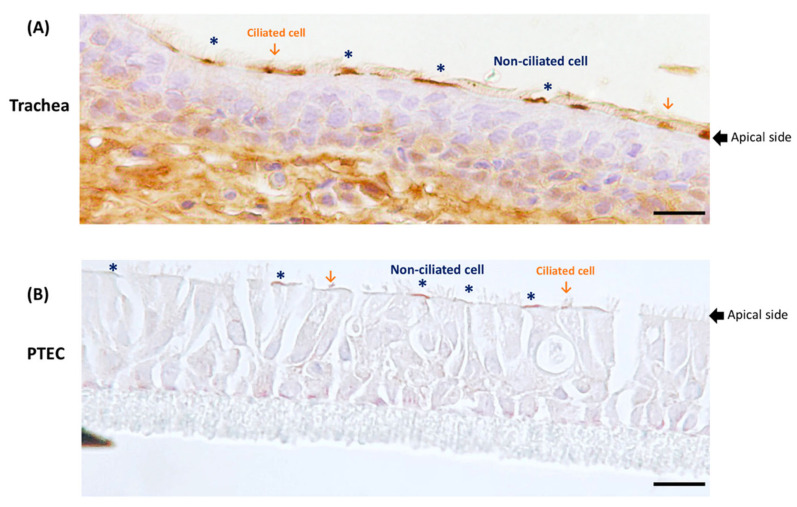
Expression of pAPN on the trachea (**A**) and well-differentiated porcine tracheal epithelial cells (wdPTECs) (**B**) by immunohistochemistry staining. (**A**) cell-selective expression of pAPN in porcine tracheal tissue; (**B**) the expression of pAPN location on wdPTECs; The presence of pAPN antigen (brown staining) detected on the apical side can be shown on both the non-ciliated of pseudostratified epithelial cells (asterisk) and the ciliated of pseudostratified epithelial cells (arrow). Scale bar = 20 µm.

For many viruses, the expression and distribution of appropriate receptors on the surface of host cells are a major determinant of the cell tropism. Therefore, we attempted to elucidate the location of pAPN on the respiratory tract. We applied immunohistochemistry (IHC) to stain pAPN on the porcine trachea and on wdPTECs (Figure 3). IHC staining of pAPN was detected in non-ciliated cells, scattered goblet cells, and occasionally on ciliated cells in the trachea (Figure 3A). A similar distribution pattern of pAPN expression was observed on wdPTECs, i.e., IHC staining of pAPN was detected mostly on non-ciliated cells, and occasionally on scattered goblet cells, and only occasionally on ciliated cells (Figure 3B). As infection by PRCoV was also detected mainly in non-ciliated cells (Figure 2), the expression of pAPN is consistent with the cell tropism of PRCoV.

### 3.4. Susceptibility to Infection and Expression of APN are Differentiation-Dependent

**Figure 4:**
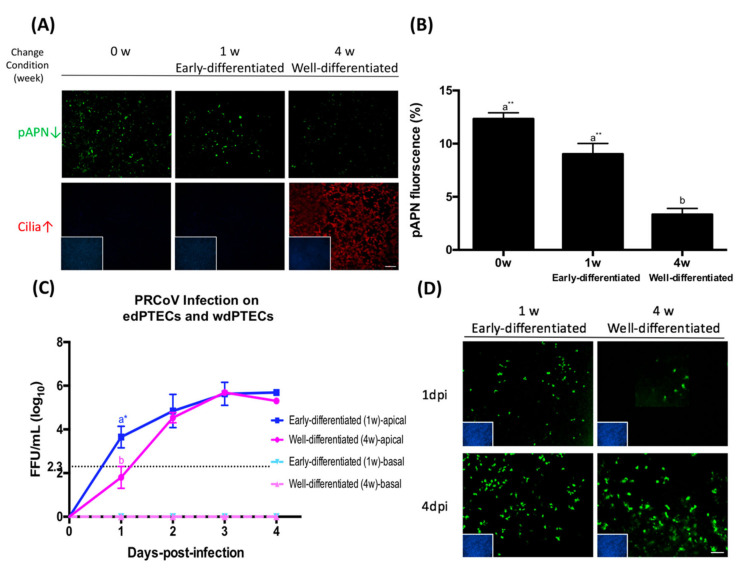
The expression of pAPN is associated with porcine tracheal epithelial cells (PTECs) differentiation. (**A**) PTECs were fixed after 0, 1, and 4 weeks of change condition, pAPN protein location in porcine airway epithelia was determined by using immunofluorescence staining for pAPN (green), β-tubulin (red), and DAPI (blue). (**B**) To quantify the expression of pAPN, the areas containing green fluorescent were determined by ImageJ; (**C**) replication of PRCoV in early differentiated (ed) and well-differentiated (wd) porcine airway epithelial cell cultures. EdPTECs and wdPTECs were inoculated with PRCoV from the apical side at 1 × 10^3^ FFU. Viruses released from the apical side were harvested at different time points and titrated by focus-forming assay in ST cells. The results were shown as means ± SEM of nine PTECs from three independent donors. Each sample was processed with two technical replicates. The dashed line was the detection limit for the foci-forming assay. Statistical analysis was performed by one-way ANOVA and followed by Tukey’s multiple comparisons test. a, b significant differences between groups are indicated with different letters (** *p* < 0.01, * *p* < 0.05). (**D**) EdPTECs and wdPTECs were fixed on 1- and 4-days post infection, respectively. EdPTECs and wdPTECs were stained for PRCoV nucleocapsid (green) and DAPI (blue). Scale bars, 25 μm.

The expression of the receptor might depend on the state of cellular differentiation [23]. To validate this notion with our in vitro model, we fixed the primary porcine tracheal epithelial cells on the filter after 0, 1, 4 weeks of incubation under air–liquid interface conditions. We applied immunofluorescence microscopy to visualize the expression of pAPN as well as the presence of cilia and used ImageJ to quantity the amount of expression of pAPN. The result is shown in Figure 4A,B. As the differentiation progressed, the number of ciliated cells increased. Parallel to the increased staining of β -tubulin, the staining of pAPN was decreased. This result indicates that pAPN is expressed more abundantly in early-differentiating cells rather than in well-differentiated cells.

Next, we analyzed whether the change in the expression of pAPN during differentiation affects the infection by PRCoV. We infected early differentiated PTECs (edPTECs) and well-differentiated PTEC (wdPTECs), respectively, with PRCoV. As shown in Figure 4C,D, PRCoV replicated more efficiently in edPTECs than in wdPTECs. This difference was evident at one-day post-infection, when the titer of infectious virus in the supernatant of edPTECs was almost 100-fold higher compared to that of wdPTECs, indicating a correlation between the expression of pAPN and susceptibility to infection by PRCoV.

### 3.5. Infection in Bronchial Cells Is More Efficient Than It Is in Tracheal Cells

**Figure 5:**
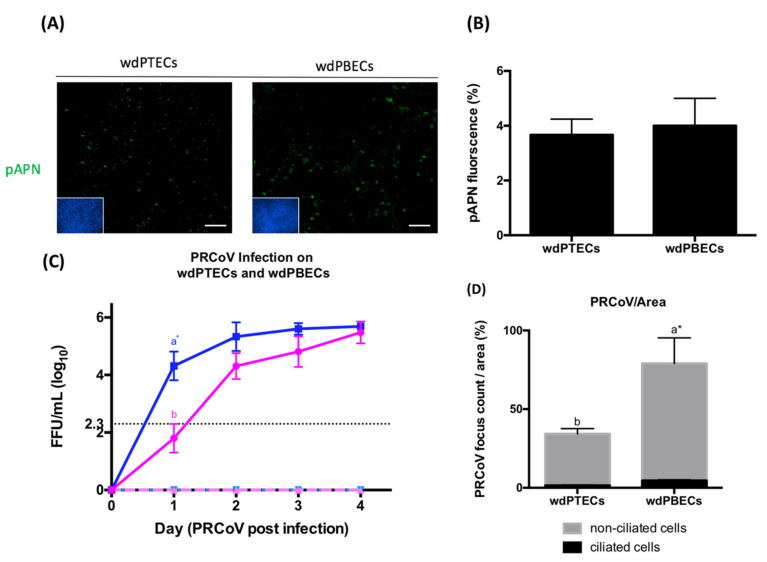
Infection in bronchial cells is more efficient than it is in tracheal cells (**A**) The expression of pAPN on well-differentiated porcine tracheal epithelial cell (wdPTECs) and well-differentiated porcine bronchial epithelial cell (wdPBECs). WdPTECs and wdPBECs was fixed on the 4 weeks of change condition, pAPN protein location in porcine airway epithelia was determined by using immunofluorescence staining for pAPN (green), DPAI (blue). (**B**) To quantify the expression of pAPN, the areas containing green fluorescent were determined by ImageJ; (**C**) Replication of PRCoV Bel85 in wdPTECs (pink) and wdPBECs (blue). WdPTECs and wdPBECs were inoculated with PRCoV from apical sides at 1 × 10^3^ FFU. Viruses released from the apical side (solid line) and basal side (dashed line) were harvested at different time points and titrated by focus-forming assay in ST cells. (**D**) Quantification of PRCoV antigen on the surface of ciliated cells and non-ciliated cells on 1-day post infection was calculated by ImageJ. The results were shown as means ± SEM. Statistical analysis was performed by one-way ANOVA and followed by Tukey’s multiple comparisons test. a, b significant differences between groups are indicated with different letters (* *p* < 0.05).

PRCoV replicates predominantly in the lungs and is mostly detected in the lower respiratory tract [21]. First, we applied immunofluorescence microscopy to visualize the expression of pAPN and used ImageJ to quantify the amount of expression of pAPN on wdPTECs and wdPBECs. The result is shown in Figure 5A,B. This result indicates that there is no significant difference between wdPTECs and wdPBECs in the expression of pAPN. To compare the replication efficiencies of PRCoV, in wdPTECs and wdPBECs, filter-grown ALI cultures were inoculated apically with PRCoV. The cell supernatants were collected from the apical and basal filter compartment at 0, 1, 2, 3, and 4-day-post-infection (dpi), respectively, and analyzed for the presence of an infectious virus. As shown in Figure 5C–E, compared to the wdPTECs, PRCoV replicated more efficiently in wdPBECs. At one-day post-infection, the infectivity determined in the supernatant of the bronchial cells was about 100-fold higher compared to the value of tracheal cells. At later time points, the differences between the virus titers continuously decreased. At 4 dpi, the amounts of infectious virus determined in the supernatants were almost the same for both wdPTECs and wdPBECs. The preferential infection of non-ciliated cells reported above (Figure 2C) was also observed with wdPBECs (Figure 5D).

## 4. Discussion

Understanding the host–pathogen interactions at the cellular and molecular level is important for attempts to control disease. We developed an ALI culture system for differentiated porcine airway epithelial cells, and inoculated PRCoV on this in vitro model. Subsequently, we used IFA or IHC staining to visualize the receptor and viral antigen. To our knowledge, this is the first time that animal coronavirus infection and its receptor distribution have been investigated in differentiated airway epithelia cells. We showed for the first time that well-differentiated cells support PRCoV replication with viral entry and egress occurring primarily from the apical surface. We report that pAPN is more abundantly expressed on the apical than on the basolateral surface of tracheal cells and PTECs, and the distribution of pAPN is correlated with the PRCoV tropism. One point of special interest was the novel observation that PRCoV infection of porcine airway epithelia was dependent upon the state of epithelial differentiation. A difference in the susceptibility to infection was also detected when tracheal and bronchial cells were compared. The increased susceptibility to infection of bronchial epithelial cells was, however, not due to an increased abundance of APN on the cell surface. Since APN is also the receptor for several other human and animal viruses, these findings are relevant also for understanding the infection by these more common pathogens.

One of the most striking findings of this study is related to the cell tropism of PRCoV. Identification of the cell types infected by PRCoV is a crucial piece of information about the pathogenesis. The results reveal that PRCoV preferentially targets non-ciliated and non-mucus-producing cells. The human coronaviruses, HCoV-229E and MERS-CoV, have also been reported to preferentially infect non-ciliated cells [23,24]. However, previous works did not further distinguish tropism between mucus- and non-mucus-producing cells. By IFA staining with Mucin-5AC, our results revealed that PRCoV preferentially targets non-mucus-producing cells. This finding might suggest the infection by PRCoV would not be easily cleared by the mucociliary clearance function. Therefore, these discoveries are consistent with the clinical implication that PRCoV only causes the mild syndrome without the loss of ciliated cells. Though non-mucus producing cells are predominantly infected, the cell tropism of PRCoV expands—though at a lower efficiency—to ciliated cells and mucus-producing cells, suggesting that these cells are also targeted by PRCoV.

The tissue distribution of APN is one of the determinants in the pathogenesis of coronavirus infections. Various in vitro models were established to investigate the expression of the receptor [25,26,27,28]. Previous studies reported that the expression of pAPN is on the apical side of polarized LLC-PK1 cells and polarized intestinal epithelial cells [26,29]. These findings are confirmed by our analysis of the trachea and wdPTECs. However, other aspects of our study cannot be elucidated with immortalized cells. In our PTEC model, we demonstrated that the expression of pAPN is readily detectable on non-ciliated cells, occasionally on ciliated cells, and to some extent on the goblet’s cells. The expression of pAPN corresponds to the cellular tropism of PRCoV. This finding is similar to the HCoV-229E. The expression of hAPN is mainly on the non-ciliated cells, and the viral tropism of HCoV-229E is mainly on the non-ciliated cells [12]. To sum up, porcine differentiated airway epithelial cells reveal the complex findings that could not be elucidated with immortalized cell lines.

The type and number of different cells in the respiratory tract varies at different stages of differentiation. In the very early phase, basal cells are predominant. At a later stage, the basal cells have started to differentiate into ciliated cells and goblet cells. When this process is finished, the airway epithelium consists of well-differentiated cells [30,31]. In our study, we demonstrated that the cells in an early differentiation stage express higher levels of pAPN, whereas well-differentiated cells express lower amounts of this protein. A different expression pattern has been described for ACE2. It is higher on well-differentiated cells than on early-differentiating cells [23,32]. It will be of great interest to investigate the APN expression in PTECs at each cell stage in the future.

The receptor is important for viral entry, but it is certainly not the only determinant [33]. In a study by Delmas et al. [33], PRCoV was found to replicate to a higher infectivity titer on pAPN-BHK cells than on parental BHK cells which can not be infected by PRCoV. In addition, pre-incubation of the pAPN-BHK cells with the anti-APN antibody G43 resulted in a complete block of PRCoV infection [33]. These findings indicate that the PRCoV infectivity is related to pAPN expression. It should be noted that APN is required for efficient virus entry. If APN is lacking or expressed at a low extent, virus infection may still occur but with low efficiency. This may explain why it replicated in well-differentiated cells more slowly but reached the same titer in the final titers. Interestingly, a difference in the susceptibility to infection was also detected when tracheal and bronchial cells were compared. Nevertheless, the difference between tracheal and bronchial cells is not due to an increased abundance of APN on the cell surface. Other factors, e.g., a supplementary receptor, a co-factor, or transmembrane serine proteases like TMPRSS2, should be considered for a potential role in viral entry [34,35]. SARS-CoV-2 uses the ACE2 for entry and the serine protease TMPRSS2 for S protein priming [36]. Epidermal growth factor receptor (EGFR) is a co-factor of TGEV, and plays a synergistic role with APN early in TGEV infection [37]. However, information about entry of PRCoV is limited.

In summary, we elucidated the pathogenesis of PRCoV on the porcine airway epithelial cells to analyze the route of infection and viral tropism. These findings are expected to also apply to coronaviruses of other species. In addition, we provided the new insights that expression of APN is dynamic and associated with cellular differentiation, a finding that may underlie susceptibility to infection. Taken together, these porcine airway epithelial cells will help us to address questions concerning emerging respiratory coronaviruses and may even provide the platform to study potentially efficacious therapeutic options.
